# Phytochemical Profile and Antioxidant, Antiproliferative, and Antimicrobial Properties of *Rubus idaeus* Seed Powder

**DOI:** 10.3390/foods11172605

**Published:** 2022-08-27

**Authors:** Giuseppe Mannino, Graziella Serio, Raimondo Gaglio, Gabriele Busetta, Lorenza La Rosa, Antonino Lauria, Luca Settanni, Carla Gentile

**Affiliations:** 1 Innovation Centre, Department of Life Sciences and Systems Biology, University of Turin, Via Quarello 15/A, 10135 Turin, Italy; 2 Department of Biological, Chemical and Pharmaceutical Sciences and Technologies, University of Palermo, Viale delle Scienze, 90128 Palermo, Italy; 3 Department of Agricultural, Food and Forest Sciences, University of Palermo, Viale delle Scienze, 90128 Palermo, Italy

**Keywords:** agricultural waste, anticancer activity, cellular oxidative stress, minimum inhibitor concentration, phytochemicals, red raspberry, sustainability

## Abstract

In the context of the contemporary research on sustainable development and circular economy, the quest for effective strategies aimed at revaluation of waste and by-products generated in industrial and agricultural production becomes important. In this work, an ethanolic extract from red raspberry (*Rubus idaeus*) seed waste (WRSP) was evaluated for its phytochemical composition and functional properties in term of antioxidative, antiproliferative, and antimicrobial activities. Chemical composition of the extract was determined by both HPLC-ESI-MS/MS and spectrophotometric methods. Phytochemical analysis revealed that flavan-3-ols and flavonols were the major phenolic compounds contained in WRSP. The extract demonstrated very high radical-scavenging (4.86 ± 0.06 µmol TE/DW) and antioxidant activity in a cell-based model (0.178 ± 0.03 mg DW/mL cell medium). The WRSP extract also exhibited antiproliferative activity against three different epithelial cancer cell lines (MCF-7, HepG2, and HeLa cells) in a dose-dependent manner. Finally, microbiological assays showed the absence of colonies of bacteria and microscopic fungi (yeasts and molds) and revealed that the WRSP extract has a large inhibition spectrum against spoilage and pathogenic bacteria, without inhibitory activity against pro-technological bacteria. In conclusion, the obtained results show that WRSP is a rich source of phytochemical compounds exerting interesting biological activities. For these reasons WRSP could find applications in the nutritional, nutraceutical, and pharmacological fields.

## 1. Introduction

Plant foods are major sources of bioactive compounds, and their consumption is linked to potential human health benefits. This awareness has led to a significant increase in global demand for fruits and vegetables. On the other hand, large amounts of waste are inexorably generated not only by plant food consumption but also by overproduction and improper storage practices [1]. For many years, this waste was used as animal feed or disposed of in landfills. Currently, due to an increased perception of the benefits of the circular economy, plant food waste and by-products are now being investigated for recycling in food and non-food applications [2,3]. In particular, agri-food waste and by-products are still rich in bioactive compounds [4,5,6] and can therefore be used as raw materials for cosmetic, nutraceutical, and pharmaceutical formulations.

The red raspberry, or European raspberry, is the fruit of *Rubus idaeus*, a member of the Rosaceae family. Although it is a species native to western Asia, it is widespread in southeastern Europe. It is an “aggregate fruit” containing several drupes originating from the pistil of each flower. Considering that each drupe contains a small seed, the whole fruit has a large number of seeds. In 2020, the global production of red raspberries was close to 900 thousand (895,771) tons [7,8]. In recent years, Italian red raspberry cultivation has significantly increased, and although fruit production is less than two thousand tons per year, marketing of these red fruits is very profitable [8]. The fruits of *R. idaeus* have been employed in traditional medicine for the treatment of wounds, renal diseases, inflammation, and infections [9]. On the other hand, scientific data show that red raspberry possesses interesting nutritional and functional value. It is a rich source of vitamin C, ranging from 5 to 40 mg per 100 g of fresh fruit, and also it contains large amounts of polyphenols. In particular, scientific data have shown a significant presence of ellagitannins, phenolic acids, and flavonoids, including anthocyanins, catechins, and proanthocyanidins [10,11]. In a simulated digestion model, Mihailovic and colleagues (2019) also showed that, under gastrointestinal conditions, red raspberry polyphenols are stable, bioaccessible and, therefore, potentially bioavailable [10]. Conversely, experimental data in both in vitro and in vivo models have demonstrated protective effects, including antioxidant, antitumor, and antidiabetic activities, of extracts of *R. idaeus* fruits [12,13,14].

The successful consumption of berries in the diet is due not only to their organoleptic properties, but also to their high nutraceutical value. Consequently, innovative strategies have recently been developed to increase their intake in the human diet [15]. Similar to other berries, red raspberry is often intended for fresh consumption and is highly valued by consumers for its taste and aroma. However, because of its pronounced perishability, the fruit is commonly destinated to industrial processing for the production of juices, liqueurs, jellies, syrups, and natural dyes. In processing, the seeds are the main by-product, and several strategies for their valorization have been explored. For example, cold pressing of the seeds has been evaluated with the aim of obtaining a highly valued oil for cosmetic and nutritional purposes [7]. However, even this practice produces an additional waste, an organic insoluble residue represented mainly by the seed coats. The functional value of cold-pressed seed powder extracts for various berries has been previously evaluated and demonstrated. In particular, these works agree that this type of food waste is really rich in bioactive compounds, including flavonoid, terpenoid, and alkaloid compounds, that may exert extremely interesting biological activity [16,17,18,19]. However, the studies on valorisation of *R. ideaeus* seed flour are rather scarce. In particular, Parry et al. (2006) have discovered the presence of high amounts of polyphenols and a significant antioxidant activity [19]. Moreover, Kang et al. (2016) have assessed inhibition of high sugar intake-mediated metabolic dysfunction in a murine model, identifying ellagic acid as the main acid responsible of the observed protective effects [20].

The aim of this work was to deepen the phytochemical profile of waste red raspberry seed powder (WRSP). Moreover, functional value, including antioxidant, anticancer, and antimicrobial activities, has been evaluated. 

## 2. Materials and Methods

### 2.1. Plant Materials and Preparation of Extracts

The raw material used for this work was obtained from a local company involved in the extraction of vegetable oils from seeds. The waste of the seed extraction process consists of the seed powder deprived of the oil fraction after a cold-pressing process. After oil extraction, the *R. idaeus* exhausted seeds were grinded in order to obtain a fine powder. The processed plant material was then stored at room temperature (RT) until extraction process. For the preparation of the extract, three different aliquots of WRSP were extracted using 70:30 (*v*/*v*) ethanol:water in 1:20 (*w*/*v*) ratio [21]. The samples were vortexed for 5 min, sonicated at RT for 20 min, and shaken for 24 h at 4 °C in the dark. Subsequently, samples were centrifuged at 2000 rpm for 10 min at 4 °C. The extraction procedure was repeated twice, and the supernatants were filtered (Millex HV 0.45 µm, Millipore, Billerica, MA, USA) and collected together. Meanwhile, the residue was extracted again with 10 mL of the same extraction solvent and the supernatant was separately stowed (exhausted). The obtained WRSP ethanolic extract was stored at −20 °C until further chemical and biological analyses.

### 2.2. Phytochemical Characterization

An Agilent Technologies 1200 liquid chromatography (LC) coupled to a Diode Array Detector (DAD) and a 6330 Series Ion Trap Mass Spectrometer (MS) System (Agilent Technologies, Santa Clara, USA) was employed for the phytochemical profile of WRPS. The separation was carried out using the chromatographic gradient and conditions previously reported [22]. Analyses were performed in triplicate.

### 2.3. Redox Active Proprieties 

Radical scavenging activity of the WRSP extract was assessed by ABTS assay, as previously reported [23]. Briefly, the green-stable cationic radical ABTS^•+^ was freshly prepared incubating ABTS salt (Sigma Aldrich, St. Louis, MO, USA) with K_2_S_2_O_8_ (VWR International, Radnor, PA, USA) at room temperature overnight. WRSP extract at different concentrations has been added with the radical previously diluted in ethanol. The reduction of the radical ABTS^•+^ was monitored spectrophotometrically at 734 nm, and the decolouration percentage (D) of the radical solution was evaluated using Equation (1):

(1) $$D=100\times \left[\frac{\left(A_{t0}-A_{t180}\right)}{A_{t0}}\right]$$

where A_to_ is the absorbance of the radical solution before the addition of the WRSP extract and A_t180_ is the absorbance after 180″ of the WRSP extract addition. The result was the average of three separate experiments. Trolox was used as standard, and the antioxidant activity of each assay was expressed as mmol of Trolox Equivalent (TE) per 100 g of DW.

### 2.4. Cell Culture

The human epithelial cell lines HeLa (human cervical cancer), MCF-7 (human breast cancer), and HepG2 (human hepatocarcinoma) were obtained from American Type Culture Collection (Rockville, MD, USA). MCF-7 cells were grown in DMEM while HeLa and HepG2 cells were grown in RPMI (VWR International, Radnor, PA, USA). Both culture medium were supplemented with 5% FBS (VWR International, Radnor, PA, USA), 2 mM L-glutamine (VWR International, Radnor, PA, USA), 50 IU/mL penicillin, and 50 μg/ mL streptomycin (VWR International, Radnor, PA, USA) and incubated in a humidified atmosphere with 5% CO_2_ at 37 °C [24]. Cells were mostly cultured in 75 cm^2^ culture flasks and were collected using trypsin-EDTA (VWR International, Radnor, PA, USA) before the 80% of confluence was reached.

### 2.5. Cellular Antioxidant Activity

The cellular antioxidant activity (CAA) assay was accomplished as previously described by Wolfe at al. [25] with some minor changes [26]. Briefly, 96-well plates were seeded with HepG2 cells in complete culture medium at the density of 6.0 × 10^4^ cells/well and incubated for 24 h. Subsequently, the cells were treated with 25 μM DCFH-DA salt (Sigma Aldrich, St. Louis, MO, USA) and the WRSP extract at different concentrations for 2 h. Ethanol concentration never exceeded 0.25% (*v*/*v*). Control and blank wells were incubated with 25 μM DCFH-DA in culture medium containing 0.25% ethanol (*v*/*v*). After the incubation time, cells were washed with PBS salt (Sigma Aldrich, St. Louis, MO, USA), then 600 μM2,2′-Azobis(2-methylpropionamidine) dihydrochloride (ABAP) (Sigma Aldrich, St. Louis, MO, USA) in Hanks′ Balanced Salt solution (HBSS) (Sigma Aldrich, St. Louis, MO, USA) was added to treated and control wells. HBSS alone was added to blank wells. The fluorescence of treated, control, and blank wells were evaluated every 5 min for 1 h by using a plate-reader at 37 °C. The area under the curve of fluorescence versus time was integrated to calculate the CAA values for treated and control wells using Equation (2):

(2) $$\mathrm{CAA}=100\times \left[100-\left(\frac{\int SA}{\int CA}\right)\right]$$

where ∫ SA is the integrated area of the sample wells and ∫ CA is the integrated area of the control wells. The antioxidant activity was expressed as CAA_50_, that is the WRSP extract concentration necessary for 50% of DCF formation inhibition and CAA_50_ was calculated from a concentration-response (CAA) curve using linear regression analysis, and it was expressed as mg of DW per mL of cell medium. The result, expressed as mg/mL of cell medium, is the mean value of three separate experiments. 

### 2.6. Antiproliferative Activity

Exponentially growing cells were used for the 3-(4,5-Dimethyl-2-thiazolyl)-2,5-diphenyl-2H-tetrazolium bromide (MTT) assay. The assay was executed as previously described [21]. Briefly, the cells were seeded into standard 96-well plates at a density depending on the doubling times of each cell line. After 24 h of incubation, the WRSP extract at appropriate concentrations (500-50 μg DW/mL cell culture medium) was added and cells were incubated for another 48 hrs. Ethanol concentration never exceeded 0.25% (*v*/*v*). Control cells were incubated with culture medium containing 0.25% ethanol (*v*/*v*). After the incubation time, 0.5 mg/mL MTT reagent (Sigma Aldrich, St. Louis, MO, USA) was added and discarded after a 3-h incubation at 37 °C. The blue formazan produced in living cells was dissolved by dimethyl sulfoxide (DMSO). The absorbance was measured in a microplate reader (UV-1900i, Shimadzu^®^, Milan, Italy) at 570 nm, and the percentage of growth (PG) with respect to control cells (untreated cells) was calculated according to Equation (3):

(3) $$PG=100\times \frac{\left(OD_{test}-OD_{tzero}\right)}{\left(OD_{ctr}-OD_{tzero}\right)}$$

where OD_test_ is the average of optical density after cell exposure to the extract for a chosen period of time; OD_tzero_ is the average of optical density before the extract addition; and OD_ctr_ is the average of optical density after the chosen period of time with no exposure of cells to treatment. The concentration needed to induce 50% growth inhibition (GI_50_) for each cell line was determined from concentration-response (PG) curves using linear regression analysis, and it was expressed as mg of DW per mL of cell medium. The results, expressed as µg/mL of cell medium, is the mean value of three separate experiments. 

### 2.7. Microbiological Characterization of WRSP

Ten grams of WRSP were transferred into a sterile bag (BagFilter^®^ 400, Interscience, Saint Nom, France) diluted with 90 mL of Ringer’s solution (Sigma Aldrich, Milan, Italy), and homogenized by the stomacher apparatus BagMixer^®^ 400 (Interscience, Saint Nom, France) at the highest speed (blending power 4) for 2 min. Homogenized WRSP was serially diluted and then plated on agar media for the enumeration of the main microbial groups belonging to the pro-technological, spoilage, and pathogenic populations following the approach of Messina et al. (2019) [27]. Briefly, the different microorganisms were inoculated as follows: total mesophilic microorganisms (TMM) on plate count agar (PCA); mesophilic lactic acid bacteria (LAB) rods and cocci on de Man-Rogosa-Sharpe (MRS) and Medium 17 (M17) agar, respectively; enterococci on kanamycin aesculin azide (KAA) agar; Pseudomonads on *Pseudomonas* agar base (PAB); members of the Enterobacteriaceae family on violet red bile glucose agar (VRGBA); coagulase-positive staphylococci (CPS) on Baird Parker (BP) supplemented with rabbit plasma fibrinogen (RPF); *Listeria monocytogenes* on *Listeria* selective agar base with SR0140E supplement; *Salmonella* spp. and *Escherichia coli* on hektoen enteric agar (HEA); and yeasts and moulds on malt agar (MA) supplemented with chloramphenicol (0.1 g/L). All media and chemicals were purchased from Microbiol Diagnostici (Uta, Italy). Plate counts were performed in triplicate.

### 2.8. Determination of Antibacterial Activity of WRSP

The ethanolic extract of WRSP was tested against bacteria of food origin. In particular, *Enterococcus mundtii*, *Fructilactobacillus sanfranciscensis*, *Latilactobacillus sakei*, *Levilactobacillus brevis*, *Lactococcus lactis*, and *Leuconostoc mesenteroides* were chosen among pro-technological species, *Brochothrix thermosphacta*, *Pseudomonas endophytica*, *Pseudomonas fluorescens*, *Pseudomonas lactis*, and *Pseudomonas poae* among spoilage bacteria, while *Acinetobacter guillouiae*, *Bacillus cereus*, *Escherichia coli*, *Listeria monocytogenes*, *Pseudomonas aeruginosa*, *Salmonella* Enteritidis, *Salmonella* Typhimurium, *Staphylococcus aureus*, and *Staphylococcus epidermidis* among bacteria responsible for human diseases. All spoilage and pathogenic bacteria were sub-cultured in Brain Heart Infusion (BHI) broth (Condalab, Madrid, Spain) incubated for 24 h at 37 °C, *En. munditii*, *Lc. lactis* and *Ln. mesenteroides* were reactivated in M17 (Microbiol Diagnostici) incubated for 24 h at 30 °C, *Lt. sakei* and *Lv. brevis* in MRS (Microbiol Diagnostici) incubated for 24 h at 30 °C, while *F. sanfranciscensis* in modified MRS [28]. The inhibitory activity of WRSP extract was tested by the well diffusion assay (WDA) [27,29] using sterile water as negative control, while streptomycin (10% *w*/*v*) was used as positive control. The inhibitory activity was evaluated after incubation at the optimal temperature for each strain and was considered positive only in case of a definite clear halo surrounding the wells. The strains sensitive to the extract were subjected to the minimum inhibitory concentration (MIC) determination by broth microdilution method in 96-well microplates [30]. Briefly, the extract was serially diluted in the optimal growth media for each strain (1:2) and their concentrations ranged between 100 and 3.125 mg/mL. After inoculation with approximately 10^6^ CFU/mL of each sensitive strain, the bacterial growth was measured using a ScanReady Microplate photometer P-800 (Life Real Biotechnology Co., Ltd., Hangzhou, China). WDA and MIC tests were carried out in triplicate.

### 2.9. Statistical Analysis 

All the statistical analyses were carried out using SPSS ver. 24 software (IBM, Armonk, New York, USA). The results were expressed as mean ± standard deviation (SD). 

## 3. Results and Discussion

### 3.1. Phytochemical Characterization

In order to determine the phytochemical profile of the WRSP extract, HPLC-DAD-MS/MS analyses were performed. Specifically, 37 different compounds were identified. In particular, fourteen were flavonols (#6, #7, #9, #10, #11, #12, #14, #15, #16, #22, #24, #25, #34, and #35), three were flavanones (#20, #27, and #37), two were flavonones (#18 and #26), three were flavanonol (#1, #2, and #19), and one was an O-methylated flavonol (#33) (Table 1). Interestingly, five flavan-3-ols (#3, #4, #5, #21, and #36), and nine proanthocyanidins (#8, #13, #17, #23, #28, #29, #30, #31, and #32) (PACs), polyphenols composed of subunits of flavan-3-ols [31,32], were also detected (Table 2). The sum of the identified compounds accounted to 1605.54 ± 44.98 mg per 100 g of DW. The major contribution to the quantification was given by flavan-3-ol family, which represented almost half (48%) of the quantified phytochemicals. In particular, #3, #4, #5, and #36 were glycosylated forms of the simplest existing flavan-3-ol, Catechin (#21). Moreover, HPLC analysis allowed the identification and quantification of a large amount of PACs (641.23 mg per 100 g DW) mostly characterized by a single C6-C4 interflavanic binding. The large presence of those polyphenols in the WRSP extract should not be surprising. Indeed, it is well known that PACs play an essential physiological role in seeds from the early stages of germination [31,33,34]. The most abundant PACs in WRSP were B-type (#8, #17, and #29). Regarding the degree of polymerization, most of the PACs were dimers (#8), representing about 68% of total identified PACs. 

Concerning the identified flavonols, representing about 45% of the total quantified polyphenols, #12 (quercetin) accounted for almost all of the weight determined for these polyphenols. On the other hand, this is one of the most widespread polyphenols in the plant kingdom, and it is involved in one of the main steps during the synthesis of most polyphenols in plants [35]. 

Regarding the conjugation of polyphenols with sugar moieties, most of the identified polyphenols were not glycosylated forms (#1, #8, #12, #13, #17, #19, #21, #22, #23, #24, #25, #27, #28, #29, #30, #31, and #32). Specifically, almost 97% of the identified polyphenols were aglycone, accounting for 1548.91 ± 96.47 mg per 100 g of DW. The remaining glycosylated polyphenols were conjugated to galactose (#3, #11, and #20), glucose (#2, #4, #14, #15, and #18), arabinose (#7), diglucoside (#10, #33, #36, and #37), rutinoside (#5, #6, #26, and #34), xylose (#9), sambubiose (#16), or glucuronic acid (#35) moiety. Scientific studies evaluating the potential bioavailability of polyphenols have frequently indicated that aglycones are poorly permeable to biological membranes [31,35,36,37]. Consequently, potential local activity of these phenols after ingestion of foods enriched with these phytochemicals can be presumed [31,36]. 

### 3.2. Antioxidant Proprieties

Oxidative species physiologically generated by aerobic metabolism are involved in different processes, such as proliferation, apoptosis, and gene expression. However, an unbalance among endogenous antioxidant defenses and reactive species production causes cellular oxidative damage associated with etiology and progression of several chronic diseases [38,39,40]. Dietary antioxidants intake contributes to preventing this unbalance. In particular, from the literature it is known that phytochemicals, such as polyphenols, are able to increase the endogenous antioxidant defenses thanks to both their redox-active properties and modulating effects on antioxidant gene expression [21]. On the other hand, the bioactivity of phytochemicals has frequently been referred to by their antioxidant properties. Indeed, by influencing cellular redox homeostasis, dietary phytochemicals can induce structural changes in redox-sensitive targets and, consequently, modulate their function [41]. 

**Figure 1:**
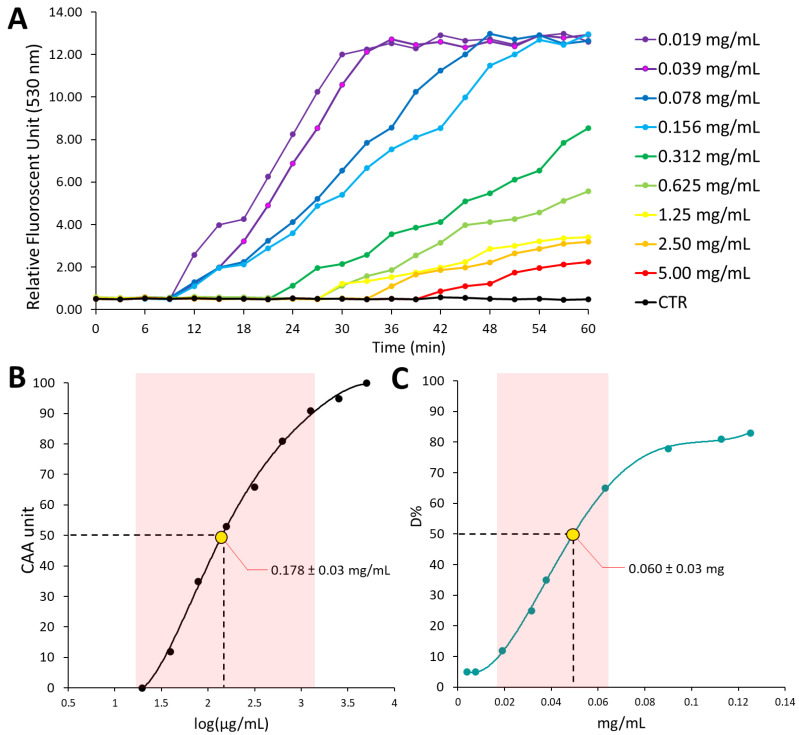
Antioxidant properties of the WRSP extract. Panel (**A**) shows the relative fluorescence of treated (mg/mL per cell medium) or untreated (control) HepG2 cells evaluated each 5 min for 1 h, after exposure to 600 µM ABA, as reported in materials and methods. Panel (**B**) shows the dose-response (CAA) curve, used to calculate CAA_50_ value, reported with the yellow dot. Panel (**C**) shows dose-response (D%) curve obtained from the decolorization of radical ABTS solution, after adding different concentrations of the WRSP extract, as reported in materials and methods. The yellow dot in Panel (**C**) shows the WRSP concentration (mg/per mL of reaction mix) necessary to 50% decolorization of the radical ABTS solution (D_50_). The red box represents the linear portion of the curve within which the CAA_50_ and D_50_ values were determined.

Different *in solution* methods have been used to estimate redox active proprieties of pure compounds, plant extracts, or biological fluids. These assays generally use radical generating systems and evaluate the ability of antioxidant compounds to scavenge synthetic free radicals. One of the most popular is the ABTS assay. Radical scavenging activity of the WRSP extract was equal to 4.86 ± 0.06 mmol TE/100 g DW (Figure 1, Panel C). With respect to the redox scavenging activity of WRSP of other berries seeds powder, the ABTS value measured for WRSP is comparable to that estimated for black currant and cranberry, meanwhile is it 2.5 and 50 folds higher than that of elder and rose hip, respectively [16,17]. In order to study whether the redox scavenging ability of WRSP extract prevent oxidative damage in a biological system, the antioxidant activity of the extract was also evaluated in a lipid peroxidation cell-based model. CAA assay not only allowed the evaluation of the antioxidant potential against peroxyl radicals but also the ability of antioxidant substances to interact with or cross membranes and their stability to cellular metabolism [42,43,44]. The estimated CAA_50_ was 0.178 ± 0.03 mg DW/mL cell medium (Figure 1, Panel A and B). This value is indicative of a relevant antioxidant activity. Moreover, this data turns out to be two orders of magnitude lower, thus indicating a greater activity, compared to that determined by Wolfe 2008 for raspberry extracts [25,42] (togliere 43). 

### 3.3. Antiproliferative Activity

**Figure 2:**
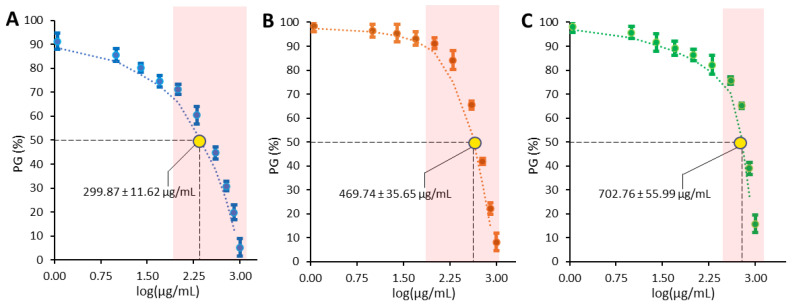
Antiproliferative activity exerted by the WRSP extract on (**A**) HepG2, (**B**) MCF-7, and (**C**) HeLa cell line. Dose/response curves are shown as percent growth (PG%) versus concentration expressed as Log(µg DW/mL cell medium). The bars reported near the coloured dots represent the standard deviation of each measurement at the relative concentration. The yellow dot reports the GI_50_ (µg DW/mL cell medium) of WRSP on each cell line. The red box represents the linear portion of inhibition curve within which the GI_50_ value was determined.

The antiproliferative potential of the WRSP extract was assessed via MTT assay, using three epithelial human tumor cell lines: cervical cancer cells (HeLa), hepatocarcinoma cells (HepG2), and breast cancer cells (MCF-7). The concentration required for 50% inhibition of cell growth (GI_50_, Growth Inhibitory 50%) was calculated by linear regression, using a concentration/percentage growth (PG) curve (Figure 2). Cells were exposed to concentrations of WRSP extract ranging from 10 to 1000 µg DW/mL of cell culture medium. The results displayed an antiproliferative activity concentration-dependent, with variability of effects in function of the cell line considered. In particular, WRSP extract showed a stronger effect on HepG2 cells (299.866 ± 11.617 µg/mL) (Figure 2, Panel A), while a progressively smaller effects was observed on MCF-7 (469.739 ± 35.646 µg/mL) (Figure 2, Panel B) and HeLa (702.760 ± 55.986) cells (Figure 2, Panel C). This difference could be a consequence of peculiar protein expression patterns in the three cell lines. On the other hand, peculiar phytochemicals in WRSP, acting on differently expressed targets in the cells, could contribute, presumably in synergistic actions, to the observed activity.

Quercetin and catechin are the main dietary polyphenols and, with dimer B-type PAC, represent the most abundant phytochemicals in the WRSP extract. In particular, the quantity of quercetin is equal to over 40% of the total weight of the identified polyphenols. Bioavailability of quercetin and its glycosylated forms has been previously demonstrated [45] and several studies attest the antiproliferative properties of these polyphenols. Moreover, induction of cell cycle arrest and apoptosis by quercetin in cancer cells has been documented [46]. On the other hand, epidemiological studies have reported that intake of quercetin-rich food reduces the risk of different types of cancer [47]. 

HPLC-DAD-MS/MS analysis revealed that WRSP is also a very rich source of PACs. Along with other health protective effects [33], anti-carcinogenic activity of proanthocyanidins from different sources have been extensively documented and the involved molecular targets identified. Although the very low bioavailability of high-molecular-weight PACs, the literature data showed that smaller oligomeric procyanidins, in particular dimers and trimers, are both stable in digestive conditions and absorbed in the gut. WRSP extract contains mainly low-molecular-weight PACs. In particular, the dimer B-Type represents over 60% of the total identified PACs in the extract [31,45,48].

It must be emphasized that the combined action of multiple polyphenols, as occurs in a complex matrix, may be higher than that of a single polyphenol. A greater cell growth inhibition activity has been demonstrated for various combinations of polyphenols when compared to the activity of the single polyphenol. This evidence may not only be the result of the ability of phytochemical mixtures to affect multiple pathways involved in cancer simultaneously but also of the possible ability of different phytochemicals to reciprocally increase their bioavailability [49]. Anticancer effects of combinations of epigallocatechin gallate (ECGC) and quercetin have been demonstrated in ovarian and prostate cancer [50] and co-adjuvant therapy efficacy of catechin and procyanidin B2 with docetaxel was demonstrated in MCF-7 cells [51]. 

### 3.4. Microbiological Characterization and Inhibitory Properties of WRSP

In order to investigate the microbiological safety of WRSP and its potential application as a functional ingredient or natural antimicrobial food additive, microbiological characterization of WRSP was performed along with the evaluation of the inhibitory properties of the WRSP extract against bacteria growth. 

The microbiological counts of WRPS did not reveal the presence of bacteria or microscopic fungi (yeasts and molds) (data not shown), highlighting the hygienic suitability of this by-product for food applications. In fact, it is well known that the presence of microorganisms in a by-product besides impairing its stability, when the by-product is used as a food additive, can endanger both consumer safety and stability of the food to which it is added [52].

The search for new antimicrobial food additives is a topic that has been attracting increasing attention over the past few years, since foodborne resistant diseases are one of the most important public health problems associated with the risk of emergence of multidrug-resistant bacterial strains in the food chain [53]. As a result, there is a raising demand for natural plant-derived preservatives because they are perceived as more natural and safe [54,55,56]. To evaluate a potential use of WRSP as food preservative, we evaluated its ability to inhibit the growth of undesirable microorganisms generally associated with foods [57]. 

The antibacterial activity of WRSP extract against spoilage and pathogenic bacteria is shown in Table 3. A considerable inhibition activity has been observed against both *Br. thermosphacta,* which is commonly associated with meat spoilage [58], and all *Pseudomonas* strains, commonly involved in the physicochemical change and generation of off-flavours in a wide range of foods of both plant and animal origin [59,60]. Regarding pathogenic bacteria, the WRSP extract was highly effective against four out of the nine tested strains, recording a diameter of the inhibition area around the wells of 13.5 mm for the *B. cereus* and of about 18 mm for *A. guillouiae*, *St. aureus,* and *St. epidermidis*. These microorganisms are relevant pathogens commonly associated with food consumption [61,62]. The antimicrobial activity of WRSP was also characterized in terms of MIC (Table 3). Only a MIC of 100 mg/mL was reached for the strain *B. cereus* ICE70, while a MIC of 25 mg/mL was observed for all the other strains. These results suggest that this extract has great potential applications as natural food preservative.

Interestingly, the development of pro-technological bacteria was not inhibited by WRSP (Table 3), indicating it is harmless against the lactic acid bacteria (LAB) used in food fermentation processes [63]. These results are very relevant because, in consideration of the previously demonstrated antioxidant and antimicrobial properties of WRSP, they indicate the suitability of WRSP as a functional or natural antimicrobial ingredient also in fermented foods.

The obtained inhibition profiles, resulting in no effects on LAB growth, are rather unexpected considering that usually antibacterial agents show greater activity against Gram-positive rather than Gram-negative bacteria. The mechanism underlying the observed activity needs further investigation. Here we can just assume that the strain-dependent resistance to WRSP components can justify the observed inhibition profiles.

## 4. Conclusions

This work demonstrates that the waste generated by the cold pressing of red raspberry seeds can be an interesting plant raw material for the extraction of bioactive compounds with different biological properties.

In particular, WRSP is shown to be really rich in several phytochemicals, including quercetin, catechin, and low weight proanthocyanidins. Further studies to elucidate the bioavailability of phytochemicals in WRSP are needed. However, considering the concentration of polyphenols in WRSP, the intake of even small quantities (5–10 g) of this plant matrix in a nutraceutical formulation or as a functionalizing ingredient can provide significant quantities of bioactive molecules with potential protective effects on human health. Therefore, antioxidant, anticancer, and antimicrobial properties evaluated and discussed in this article suggest a potential use of this agro-food waste in both food and non-food applications.

## Figures and Tables

**Table 1:** Qualitative and quantitative phytochemical characterization of flavanonols, flavonols, flavonones, flavanones, and o-methylated flavonols in the WRSP extract. Results (mg per 100 g of FW) are expressed as mean ± SD of three different experiments.

#	RT [Min]	m/z	MS/MS			CAS-ID	Chemical Formula	Compound(s)	mg/100 g
**Flavanonols**									
**1**	10.7	287				480-20-6	C_15_H_12_O_6_	Dihydrokaempferol	12.74 ± 0.73
**2**	13.6	481	319			n.a.	C_21_H_22_O_13_	Dihydromyricetin-3-O-glucoside	4.38 ± 0.11
**19**	28.2	319				27,200-12-0	C_15_H_12_O_8_	Dihydromyricetin	64.15 ± 3.96
**Flavonols**									
**6**	15.7	625	479	463	317	41,093-68-9	C_27_H_30_O_17_	Myricetin-rutinoside	0.95 ± 0.06
**7**	22.2	433	301			572-30-5	C_20_H_18_O_11_	Quercetin-3-O-arabinoside	5.25 ± 0.07
**9**	23.2	433				549-32-6	C_20_H_18_O_11_	Quercetin-3-O-xyloside	3.83 ± 0.23
**10**	24.2	625	463	301		6892-74-6	C_27_H_30_O_17_	Quercetin-3,7-O-diglucoside	3.11 ± 0.09
**11**	24.4	463	301			482-36-0	C_21_H_20_O_12_	Quercetin-3-O-galactoside	0.44 ± 0.01
**12**	24.8	301				117-39-5	C_15_H_10_O_7_	Quercetin	697.84 ± 16.81
**14**	25	447	285			482-35-9	C_21_H_20_O_12_	Kaempferol-3-O-glucoside	0.46 ± 0.02
**15**	25	463	301			482-35-9	C_21_H_20_O_12_	Quercetin-3-O-glucoside	0.71 ± 0.02
**16**	26.4	477	176			22,688-79-5	C_21_H_18_O_13_	Quercetin-3-O-glucoronide	2.44 ± 0.13
**22**	31.1	317				529-44-2	C_15_H_10_O_8_	Myricetin	18.23 ± 0.75
**24**	32.7	285				520-18-3	C_15_H_10_O_6_	Kaempferol	5.05 ± 0.27
**25**	33.9	609	463	447	301	153-18-4	C_27_H_30_O_16_	Quercetin-3-O-rutinoside	0.48 ± 0.03
**34**	45.6	593	447	431	285	17,650-84-9	C_27_H_30_O_15_	Kaempherol-3-O-rutinoside	4.82 ± 0.28
**35**	45.6	595	294			83,048-35-5	C_26_H_28_O_16_	Quercetin-3-O-sambunioside	2.42 ± 0.05
**Flavonones**									
**18**	27.7	447	285			20,344-46-1	C_21_H_20_O_11_	Luteolin-3-O-glucoside	6.32 ± 0.31
**26**	35.2	285				491-70-3	C_15_H_10_O_6_	Luteolin	2.32 ± 0.14
**Flavanones**									
**20**	29.4	451	289			20,344-46-1	C_21_H_22_O_10_	Naringenin-3-O-galactoside	2.17 ± 0.11
**27**	36.4	287				552-58-9	C_21_H_22_O_11_	Eriodictyol	5.14 ± 0.26
**37**	57.8	595	433	271		n.a.	C_27_H_32_O_15_	Naringenin-3,7-O-diglucoside	5.66 ± 0.12
**O-methylated flavonol**									
**33**	44.6	639	477	315		n.a.	C_28_H_32_O_17_	Isohermentin-3,7-O-diglucoside	1.33 ± 0.07

RT: Retention Time; m/z: mass-to-charge ratio; MS/MS: detected fragmentations; CAS-ID: Chemical Abstracts Service Identification Number; n.a.: not available.

**Table 2:** Qualitative and quantitative phytochemical characterization of flavan-3-ols and proanthocyanidins in WRSP extract. Results (mg per 100 g of FW) are expressed as mean ± SD of three different experiments.

#	RT [Min]	M-H	MS/MS			CAS-ID	Chemical Formula	Compound(s)	mg/100 g
**Flavan-3-ols**									
**3**	14.2	451	289			n.a.	C_21_H_24_O_11_	Catechin-3-O-galactoside	0.75 ± 0.03
**4**	14.9	451	289			n.a.	C_21_H_24_O_11_	Catechin-3-O-glucoside	1.32 ± 0.03
**5**	15.6	597	451	435	289	n.a.	C_27_H_34_O_15_	Catechin-3-O-rutinoside	7.34 ± 0.18
**21**	29.7	289				154-23-4	C_15_H_14_O_6_	Catechin	122 ± 3.48
**36**	51.2	613	451	289		n.a.	C_27_H_34_O_16_	Catechin-3,7-O-diglucoside	2.46 ± 0.14
**Proanthocyanidins**									
**8**	22.8	577	289			29,106-49-8	C_30_H_26_O_12_	Dimer B-Type PAC	399.61 ± 5.95
**13**	24.9	865	575	289		65,085-09-8	C_45_H_38_O_18_	Trimer A-Type PAC	14.26 ± 0.17
**17**	27.5	1153	867	577		n.a.	C_60_H_52_O_24_	Tetramer B-Type PAC	12.63 ± 0.41
**23**	31.8	1439	1437	1151	575	n.a.	C_75_H_60_O_30_	Pentamer A-Type PAC	56.16 ± 3.83
**28**	37.2	2012	1151	863	289	n.a.	C_90_H_72_O_36_	Esamer A-Type PAC	12.05 ± 0.5
**29**	37.2	2014	1153	861	577	n.a.	C_90_H_78_O_36_	Esamer B-Type PAC	5.16 ± 0.17
**30**	37.5	1437	1151	863	575	n.a.	C_75_H_60_O_30_	Pentamer A-Type PAC	30.88 ± 1.58
**31**	38.8	1151	863	575	289	n.a.	C_60_H_48_O_24_	Tetramer A-Type PAC	13.35 ± 0.64
**32**	42.5	1441	1155	865	289	n.a.	C_75_H_65_O_30_	Pentamer B-Type PAC	77.33 ± 3.16

RT: retention time; m/z: mass-to-charge ratio; MS/MS: detected fragmentations; CAS-ID: Chemical Abstracts Service Identification Number; n.a.: not available.

**Table 3:** Antibacterial activity of the ethanolic extract of WRSP.

Species	Strains	Source of Isolation	Inhibition (mm)	MIC (mg/mL)
**Pro-technological**				
*En. mundtii*	WFE3	Wheat flours	-	n.d.
*F. sanfranciscensis*	SD22	Sourdough	-	n.d.
*Lt. sakei*	SP255	Salami	-	n.d.
*Lv. brevis*	SD70	Sourdough	-	n.d.
*Lc. lactis*	CAG4	Curd	-	n.d.
*Ln. mesenteroides*	MISE643	Raw ewe’s milk	-	n.d.
**Spoilage**				
*Br. thermosphacta*	SP10	Pork meat	23.0 ± 0.4	25
*P. fluorescens*	4G628	Ready to eat salad	19.3 ± 0.2	25
*P. lactis*	SP198	Salami	17.8 ± 0.2	25
*P. poae*	4G558	Ready to eat salad	19.0 ± 0.4	25
**Pathogenic**				
*A. guillouiae*	ICE24	Food ice cubes	18.2 ± 0.3	25
*B. cereus*	ICE70	Food ice cubes	13.5 ± 0.2	100
*E. coli*	PSL52	PDO Pecorino Siciliano cheese	-	n.d.
*L. monocytogenes*	13BO	Gorgonzola cheese	-	n.d.
*P. aeruginosa*	PSA68	Animal tissue	-	n.d.
*S.* Enteritidis	ATCC13076	Unknown	-	n.d.
*S.* Typhimurium	50432	Molluscs	-	n.d.
*St. aureus*	ATCC33862	Unknown	17.8 ± 0.2	25
*St. epidermidis*	ICE244	Food ice cubes	18.6 ± 0.1	25

Results indicate the mean value of three independent assays. Abbreviations: MIC, minimum inhibitory concentration; *En*., *Enterococcus*; *F*., *Fructilactobacillus* *Lt*., *Latilactobacillus*; *Lv*., *Levilactobacillus*; *Lc.*, *Lactococcus*; *Ln*., *Leuconostoc*; *Br*., *Brochothrix*; *P.*, *Pseudomonas*; *A*., *Acinetobacter*; *B*., *Bacillus*; *E*., *Escherichia*; *L*., *Listeria*; *S*., *Salmonella*; *St.*, *Staphylococcus;* n.d., not determinated. Symbols:—no inhibition found.

## Data Availability

Data is contained within the article.
